# Generalized Connective Tissue Disease in *Crtap-/-* Mouse

**DOI:** 10.1371/journal.pone.0010560

**Published:** 2010-05-11

**Authors:** Dustin Baldridge, Jennifer Lennington, MaryAnn Weis, Erica P. Homan, Ming-Ming Jiang, Elda Munivez, Douglas R. Keene, William R. Hogue, Shawna Pyott, Peter H. Byers, Deborah Krakow, Daniel H. Cohn, David R. Eyre, Brendan Lee, Roy Morello

**Affiliations:** 1 Department of Molecular and Human Genetics, Baylor College of Medicine, Houston, Texas, United States of America; 2 Department of Orthopaedics and Sports Medicine, University of Washington, Seattle, Washington, United States of America; 3 Shriners Hospitals for Children, Portland, Oregon, United States of America; 4 Center for Orthopaedic Research, University of Arkansas for Medical Sciences, Little Rock, Arkansas, United States of America; 5 Department of Pathology, University of Washington, Seattle, Washington, United States of America; 6 Department of Medicine, University of Washington, Seattle, Washington, United States of America; 7 Medical Genetics Institute, Cedars-Sinai Medical Center, David Geffen School of Medicine at University of California Los Angeles, Los Angeles, California, United States of America; 8 Howard Hughes Medical Institute, Houston, Texas, United States of America; National University of Singapore, Singapore

## Abstract

Mutations in *CRTAP* (coding for cartilage-associated protein), *LEPRE1* (coding for prolyl 3-hydroxylase 1 [P3H1]) or *PPIB* (coding for Cyclophilin B [CYPB]) cause recessive forms of osteogenesis imperfecta and loss or decrease of type I collagen prolyl 3-hydroxylation. A comprehensive analysis of the phenotype of the *Crtap-/-* mice revealed multiple abnormalities of connective tissue, including in the lungs, kidneys, and skin, consistent with systemic dysregulation of collagen homeostasis within the extracellular matrix. Both *Crtap-/-* lung and kidney glomeruli showed increased cellular proliferation. Histologically, the lungs showed increased alveolar spacing, while the kidneys showed evidence of segmental glomerulosclerosis, with abnormal collagen deposition. The *Crtap-/-* skin had decreased mechanical integrity. In addition to the expected loss of proline 986 3-hydroxylation in α1(I) and α1(II) chains, there was also loss of 3Hyp at proline 986 in α2(V) chains. In contrast, at two of the known 3Hyp sites in α1(IV) chains from *Crtap-/-* kidneys there were normal levels of 3-hydroxylation. On a cellular level, loss of CRTAP in human OI fibroblasts led to a secondary loss of P3H1, and vice versa. These data suggest that both CRTAP and P3H1 are required to maintain a stable complex that 3-hydroxylates canonical proline sites within clade A (types I, II, and V) collagen chains. Loss of this activity leads to a multi-systemic connective tissue disease that affects bone, cartilage, lung, kidney, and skin.

## Introduction

The *Crtap* gene encodes cartilage-associated protein (CRTAP), a resident protein of the rough endoplasmic reticulum (rER) that can form a trimeric complex with prolyl 3-hydroxylase 1 (P3H1, also known as Leprecan1 and encoded by *LEPRE1*) and Cyclophilin B (CYPB, encoded by *PPIB*) [1]. The complex is responsible for the 3-hydroxylation of specific prolyl residues (Pro986) in the proα1 chains of both type I and II procollagen [1]. This enzymatic modification is catalyzed by the Fe^++^ and 2-oxoglutarate-dependent dioxygenase domain which is present at the C-terminal portion of P3H1 [2]. Although CRTAP is not enzymatically active, it is required *in vivo* for proper collagen prolyl 3-hydroxylation to take place [1]. Cyclophilin B, the other member of the complex, has peptidyl-prolyl cis-trans isomerase (PPIase) activity and is thought to facilitate the molecular winding of the collagen triple helix. Importantly, recent studies have demonstrated that the CRTAP/P3H1/CYPB complex also has chaperone activity in the rER [3]. The trimeric complex was shown to be active in two independent chaperone assays, to have PPIase activity and, like HSP47, to interact with folded triple-helical collagen, perhaps to inhibit intracellular collagen fibril formation [3].

Mice lacking both copies of the *Crtap* gene have a severe osteochondrodysplasia with rhizomelia and osteoporosis. At the tissue level, *Crtap-/-* mice have normal numbers of osteoblasts that deposit very little osteoid. Collagen fibrillogenesis is affected in that there is an increased diameter of collagen fibrils in the skin [1]. The phenotype of the *Crtap-/-* mice led to the identification of *CRTAP* mutations in patients with recessively inherited forms of osteogenesis imperfecta (OI). The severity of this form of OI disease varies based upon the nature of the *CRTAP* mutations [1], [4]. Subsequently, mutations in the *LEPRE1* gene, that encodes prolyl 3-hydroxylase 1, the second component of the rER-resident complex, were identified in patients who had no mutations in type I collagen genes or *CRTAP* [5]. Several reports followed describing additional novel *CRTAP*, *LEPRE1*, and now also *PPIB* mutations in patients with severe recessive forms of OI from different parts of the world [6], [7], [8], [9], [10]. The majority of *CRTAP* or *LEPRE1* reported mutations are null alleles associated with severe phenotypes. There are only a handful of patients, most with missense mutations, who survive childhood.

OI, whether dominant or recessive, is a generalized connective tissue disorder which can present with a variety of signs that include early osteoporosis, dentinogenesis imperfecta, hearing loss, blue sclerae, scoliosis, ligament and skin laxity, and growth deficiency [11], [12]. All affected tissues express high levels of type I collagen. Some features of OI, such as abnormal pulmonary function, have been explained as a consequence of multiple rib fractures and/or orthopedic complications of the spine (scoliosis, kyphosis and vertebral compressions) that lead to poor pulmonary ventilation and cause a progressive decrease in cardio-respiratory fitness [13]. However, the involvement of extra-skeletal tissues in the OI disease process could also be explained by nonstructural functions played by type I collagen in these organs. Alternatively, especially in cases of recessive forms of OI with mutations in members of the prolyl 3-hydroxylation complex, other collagen types may not be properly processed. Basement membrane collagens are more heavily modified by prolyl 3-hydroxylation and decreased hydroxylation could in theory lead to a multi-systemic phenotype. To explore these hypotheses, we conducted a thorough histological evaluation of extra-skeletal tissues in *Crtap-/-* mice. We have identified abnormalities affecting the lung, kidney, and skin to provide a more complete understanding of the pathophysiology of recessive OI that should guide a rational clinical assessment of these patients and may identify alternative therapeutic targets.

## Results

### *Crtap* expression in extra-skeletal tissues

**Figure 1:**
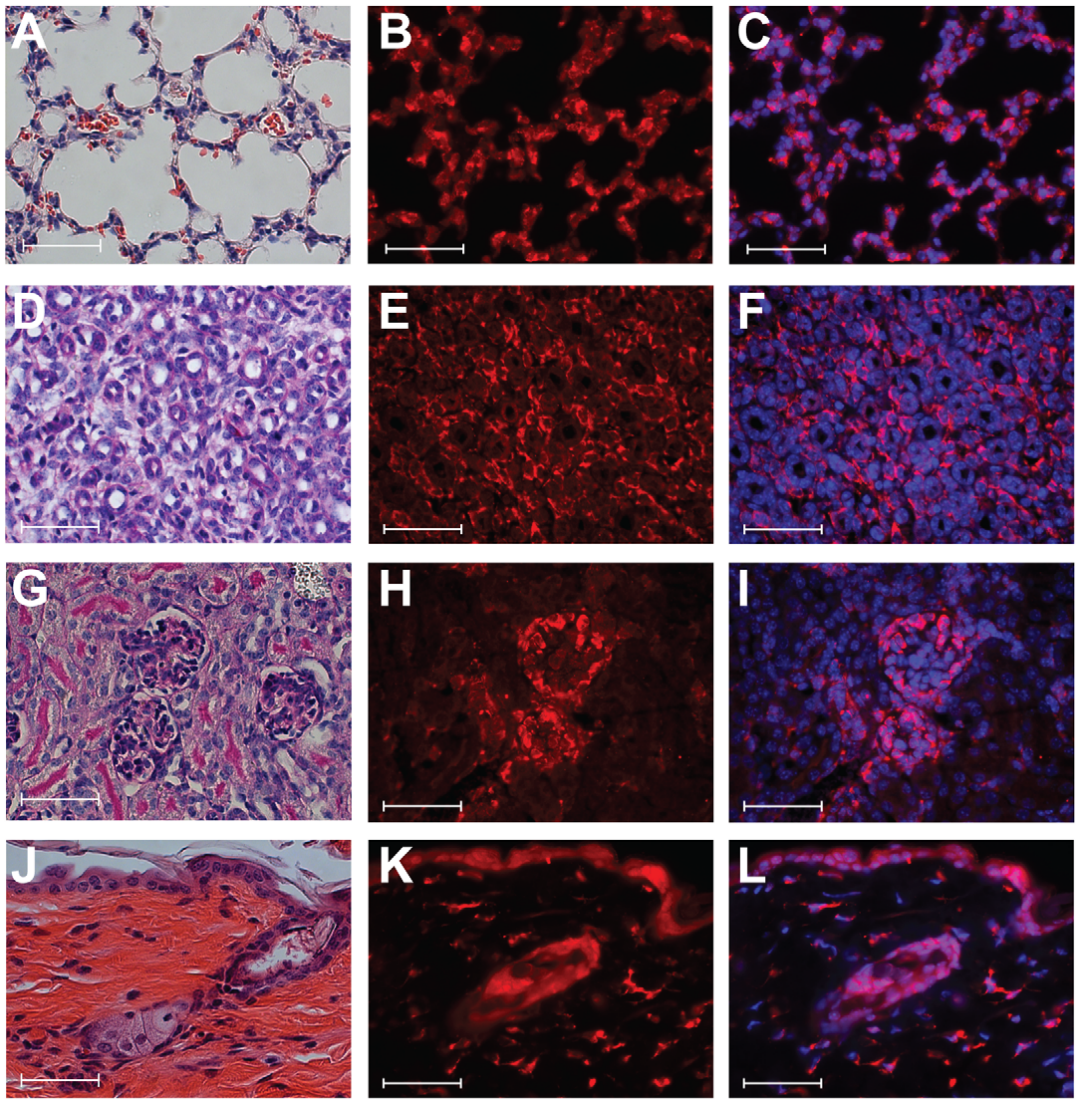
Immunofluorescence staining of CRTAP protein in wild-type mouse tissues. (**A**) Lung H&E stained section. (**B**) CRTAP protein expression in all pneumocytes. (**C**) Merge of CRTAP with DAPI. (**D**) Kidney medulla H&E stained section. (**E**) CRTAP protein seems to localize to peritubular capillaries. (**F**) Merge of CRTAP with DAPI. (**G**) H&E stained section of kidney glomeruli. (**H**) CRTAP protein is in visceral and parietal epithelial cells of the glomerulus. (**I**) Merge of CRTAP with DAPI. (**J**) Skin H&E stained section, including hair follicle. (**K**) CRTAP protein is seen as intense foci in fibroblasts distributed throughout the dermis. Epidermal and follicular staining is consistent with background staining seen in *Crtap-/-* skin stained with anti-CRTAP antibody (data not shown). (**L**) Merge of CRTAP with DAPI. All pictures are at 40× magnification, and scale bars are 50 micrometers. Specificity of staining was confirmed in all cases by using the same CRTAP antibody onto *Crtap-/-* tissue sections (data not shown).

Based on Northern blot analysis, we showed that *Crtap* is expressed in most murine tissues [14] and characterized the distribution of the protein in all components of the skeleton [1]. Here we extended these studies to non-skeletal tissues including those that do not express high levels of fibrillar collagens. We identified *Crtap* expression in postnatal lung, kidney and skin from wildtype mice. CRTAP was found throughout the lung parenchyma principally in all pneumocytes (**Figure 1B–C**). In the metanephric kidney, CRTAP was found in both visceral (podocytes) and parietal epithelial cells of the glomerulus. In addition, throughout the kidney, including the pelvis, there was interstitial staining which appears to localize to peritubular capillaries (**Figure 1E–F, H–I**). In skin there was generalized staining of the dermal fibroblasts and within blood vessels (**Figure 1K–L**). The localization of the CRTAP protein in multiple tissues extends the previous mRNA expression studies, identifies the cells in which the protein is produced and, unexpectedly, showed that CRTAP is present in tissues not particularly rich in types I or type II collagen. This last finding, in combination with the previously described broad expression patterns of P3H1 and CYPB [2], [15], [16], suggests that CRTAP, within the described ternary complex, may play a role in the prolyl 3-hydroxylation of other types of collagens. At the same time, we cannot exclude novel functions of CRTAP which could be unrelated to collagen prolyl 3-hydroxylation in these tissues.

### Tissue alterations in *Crtap-/-* mice

**Figure 2:**
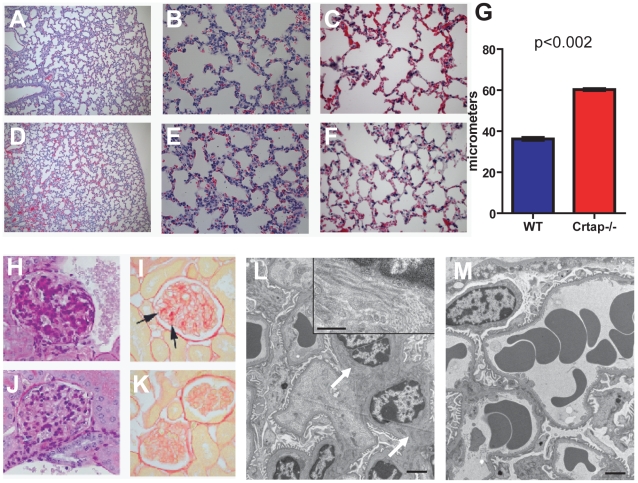
Lung and kidney abnormalities in *Crtap-/-* mice. Mutant lung H&E stained sections at P10, at 10× magnification (**A**) and 40× magnification (**B**), and in the adult at 40× magnification (**C**). Wildtype lung H&E stained sections at P10, at 10× magnification (**D**) and 40× magnification (**E**), and in the adult at 40× magnification (**F**). (**G**) Quantification of alveolar airway space by Mean Linear Intercept (MLI) shows increased airway space in *Crtap-/-* mouse lungs. Mutant adult kidney stained sections shows that some glomeruli have abnormal staining by PAS (**H**) and picrosirius red (**I**) at 40× magnification, suggestive of focal glomerulosclerosis. These changes were never observed in corresponding wildtype sections (**J–K**). Transmission EM images of *Crtap-/-* and WT glomeruli (**L–M**, scale bar  = 2 µm) showing mesangial hyperplasia and abnormal collagen fibril deposition in the mutant (arrows and inset, scale bar in inset is 0.5 µm).

**Figure 3:**
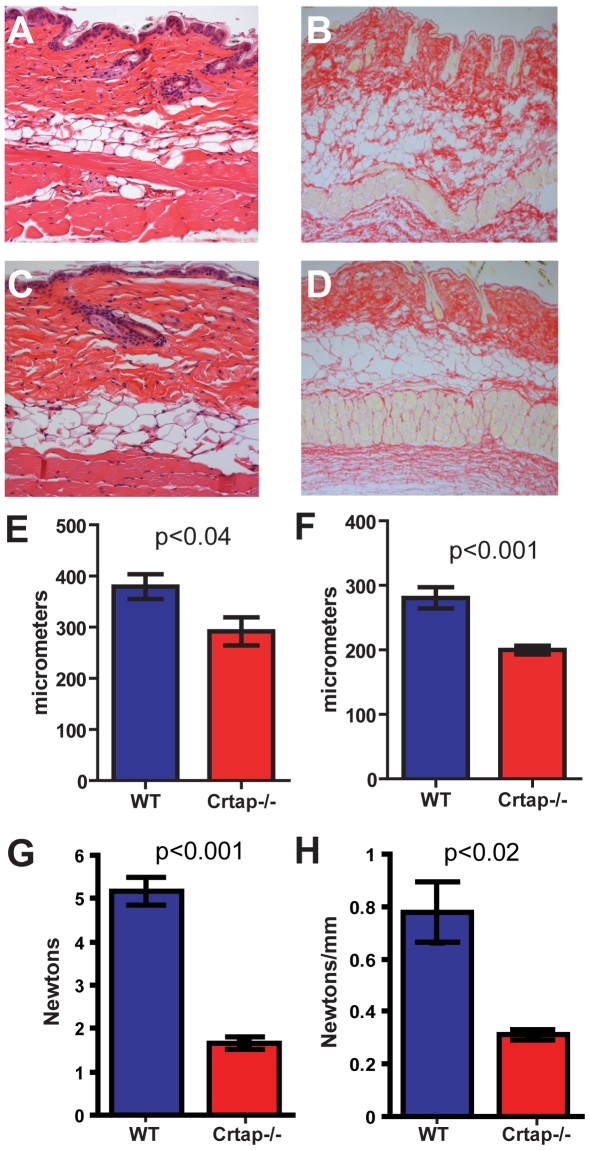
Skin defects in *Crtap-/-* mice. (**A**) Adult *Crtap-/-* skin H&E section at 20X shows thinning of the dermis relative to wildtype (**C**). (**B**) Adult *Crtap-/-* skin picrosirius red section at 20X shows disorganization of tissue layers and ECM in contrast to wildtype (**D**). Quantification of adult total skin thickness (**E**), measured from epidermis to bottom of adipose layer, and adult dermis thickness (**F**) demonstrates thinning of *Crtap-/-* skin. Significant decrease in measurements of skin peak load (G) and stiffness (H) in *Crtap-/-* skin compared to wild-type controls.

**Figure 4:**
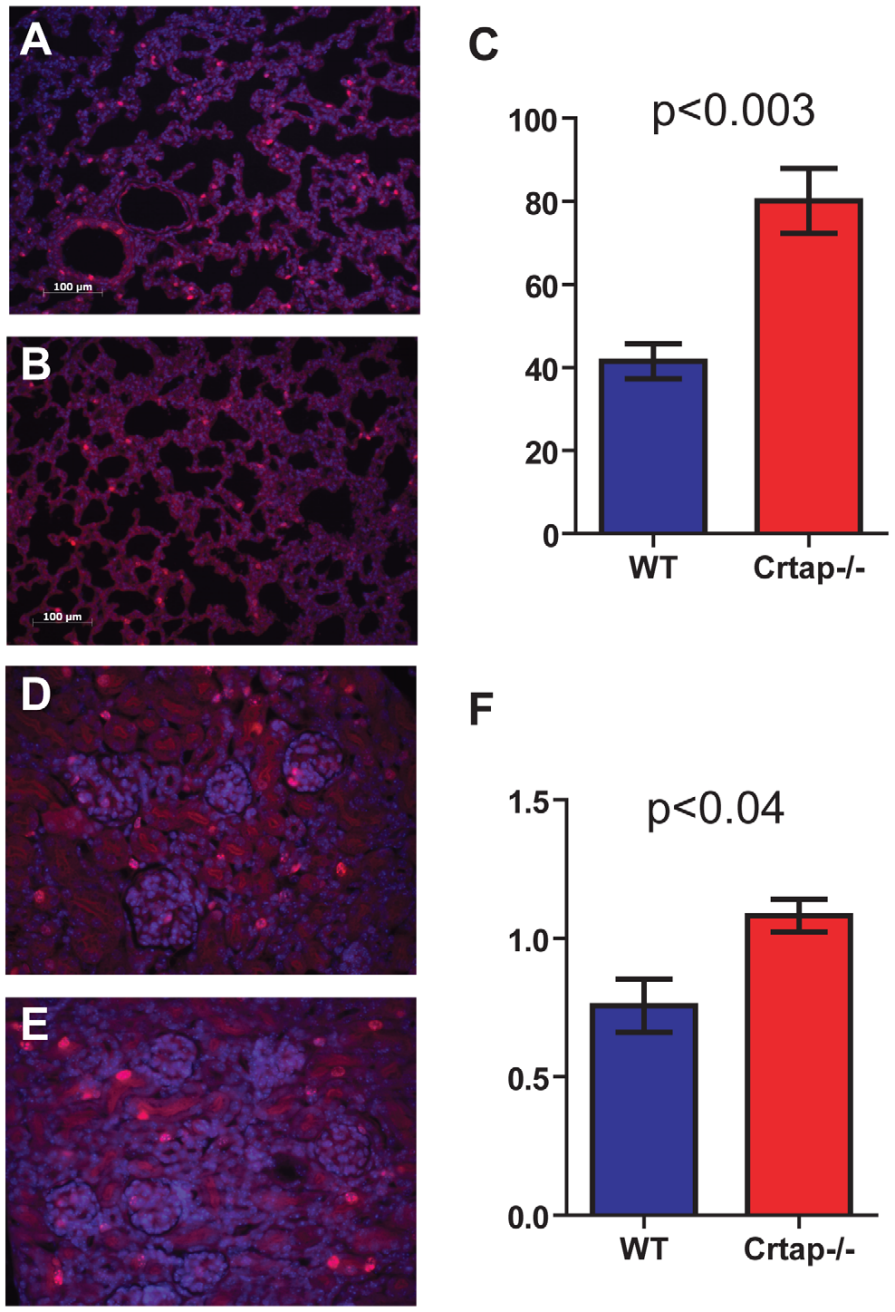
*Crtap-/-* mice have increased cell proliferation in lung and kidney. BrdU staining of *Crtap-/-* (**A**) and wildtype (**B**) lung at P10 and 20× magnification, n = 5. (**C**) Quantification of BrdU positive cells per field shows a significantly increased number of dividing cells in *Crtap-/-* lung compared to wildtype. BrdU staining of *Crtap-/-* (**D**) and wildtype (**E**) kidney at P10 and 20× magnification. (**F**) Quantification of BrdU positive cells per glomerulus shows increased number of dividing cells in *Crtap-/-* glomeruli compared to wildtype.

To identify the effects of loss of the protein, we evaluated target tissues in *Crtap-/-* mice at neonatal (P10) and adult time points (5–9 month-old). In *Crtap-/-* lungs, there was a diffuse increase in alveolar airway space often accompanied by a thinning of the alveolar walls (**Figure 2A–C**). This was already visible at P10 and became more obvious in the adult lung, as assessed by an increased Mean Linear Intercept (MLI) (**Figure 2G**). In the kidney we saw no differences at P10 (data not shown). However, in the adult *Crtap-/-* kidney we found that some glomeruli had abnormal PAS and picrosirius red staining compared with WT controls (**Figure 2H–K**). The picrosirius red, specific for collagen, revealed areas of intense staining within some glomeruli, consistent with a focal glomerulosclerosis that were never observed in WT kidney sections. These alterations were correlated with mesangial cell hyperplasia and with collagen fibril deposition as seen by transmission EM (**Figure 2L–M and inset**). The skin of *Crtap-/-* mice was notably lax, a property noted when handling the animals (data not shown). The skin was thin, the layers were disorganized, and collagen fibrils abnormal (**Figure 3A–D**). The decreased thickness of the skin of adult mice reflected a dramatic decrease in dermal thickness rather than in the adipose layer (**Figure 3 E-F-**). The mechanical properties of the skin were also examined by using a load-to-failure technique. Consistent with the gross laxity and the decreased thickness, the *Crtap-/-* skin tolerated significantly less load and was less stiff than the wildtype skin (**Figure 3 G–H**). It is known that defects in extracellular matrix proteins such as type I collagen are correlated with changes in the proliferation and survival of surrounding cells [17]. Therefore, to determine if there is an altered cellular phenotype which might result from and/or contribute to the observed histological alterations, we studied cellular proliferation and apoptosis in the affected tissues at P10. While there was no statistical difference in the number of BrdU positive cells observed in the skin (data not shown), the lungs of *Crtap-/-* mice showed a statistically significant increase of BrdU positive cells compared to WT controls (N = 5 mice, p<0.003) (**Figure 4A–C**). In P10 *Crtap-/-* kidney, there were similar numbers of BrdU positive cells in the tubular compartment; however, the number of BrdU positive cells within the glomerular compartment was increased in the *Crtap-/-* mice compared to WT controls (N = 5 mice, p<0.04) (**Figure 4D–F**). There was no difference in apoptosis between the tissues from the two genotypes (data not shown). These data show that the altered histological phenotype in *Crtap-/-* lung and kidney was correlated with evidence of increased cellular proliferation, which may be a result of disrupted extracellular matrix regulation. Alternatively, the abnormal increase in cell proliferation in these tissues could be indicative of defective terminal differentiation and synthesis of extracellular matrix proteins such as type I collagen.

### Collagen prolyl 3-hydroxylation in *Crtap-/-* tissues

**Figure 5:**
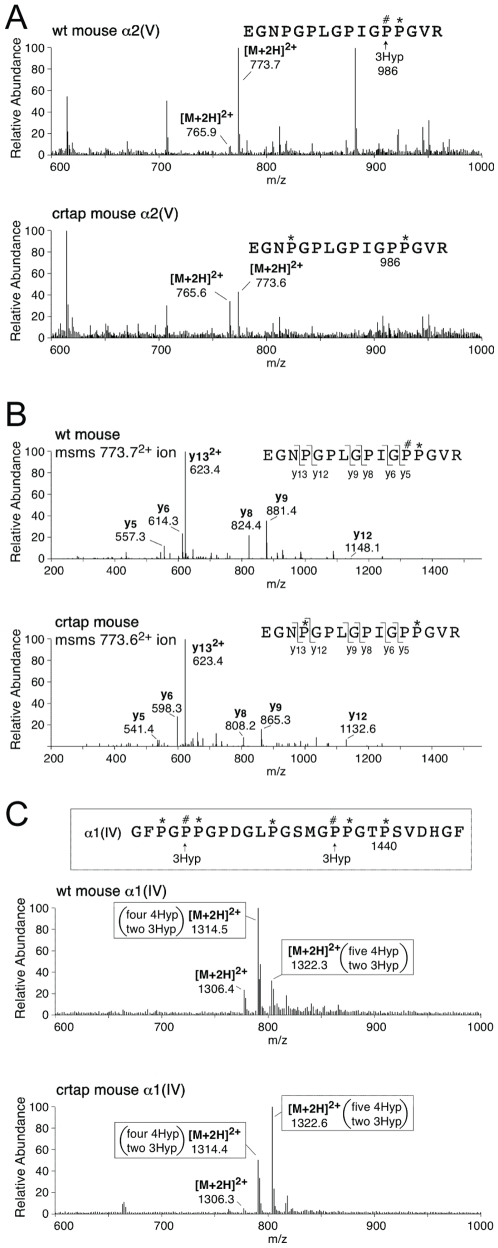
Tandem mass spectra identifies missing 3Hyp in *Crtap-/-* mouse at α2(V) P986. (**A**) Full scan spectra over the LC elution window of the tryptic peptide containing P986 from wildtype and *Crtap-/-* α2(V) from bone. (**B**) MS/MS spectra that identify the main forms of the variably hydroxylated peptides. From wildtype, P986 is almost fully 3-hydroxylated and the earlier P at 978 is not 4-hydroxylated, whereas from *Crtap-/-* P986 is not 3-hydroxylated and P978 is about 55% 4-hydroxylated. (The 765.6^2+^ ion in A is the version lacking P978 4Hyp.) (**C**) Mass spectral analysis of a 3Hyp-containing peptide from mouse kidney type IV collagen. Two full scan spectra are shown over the LC elution window of the α1(IV) tryptic peptide of sequence shown. This peptide from near the C-terminus of the main triple-helix (residue 1440 from NCBI mouse database) reveals two sites of 3Hyp that are heavily occupied in both wt and *Crtap-/-* a1(IV). MS/MS spectra (not shown) established the identities of the main post-translational variants for the labeled ions.

One of the consequences of loss of *Crtap* is the near complete absence of prolyl 3-hydroxylation at proline 986 in α1(I) and α1(II) chains of type I and II collagens [1]. While this proline is 95% 3-hydroxylated in α1(I) and α1(II) chains from WT mice, it is <1% 3-hydroxylated in *Crtap-/-* mice. The homologous proline at P986 in the α2(V) chain of type V collagen from bone and skin was 98–100% 3-hydroxylated in the WT, but <1% from bone and skin in *Crtap-/-* mice (**Figure 5A–B**), indicating that type V procollagen is a substrate for the CRTAP complex. Of note, the α1(I) chain from the kidney also lacked 3-Hyp at P986 (results not shown).

Type IV collagen is known to have a high level of prolyl 3-hydroxylation (10–15% of residues) in normal tissues. At two sites in the proα1(IV) chain, known to be highly 3-hydroxylated in bovine tissues [18], the hydroxylation of the target prolyl residues in type IV collagen from kidney in WT and *Crtap-/-* mice were similar (**Figure 5C**). These findings indicate that prolyl 3-hydroxylation of some collagens does not depend on the presence of CRTAP or is carried out by an independent system.

### Coordinated loss of both CRTAP and P3H1 proteins when either gene is mutant in human primary fibroblasts

Because P3H1 enzymatic activity requires the presence of CRTAP in both mice and humans [1], and a similar clinical phenotype is seen in patients with mutations in either *CRTAP* or *LEPRE1* [4], [5], [6], [7], [8], [9], we searched for a common mechanism of disease caused by mutations in the two human genes. CRTAP (**Figure S1A**) and P3H1 (**Figure S1C**) are both localized to the perinuclear region of the cell in normal primary human fibroblasts consistent with localization in the rough endoplasmic reticulum, as previously reported [1].

CRTAP protein was not detectable in cultured human fibroblasts derived from a patient with a homozygous frameshift mutation in *CRTAP*, c.24_31del [6] that leads to nonsense mediated decay of the mRNA (**Figure S1E**). P3H1 protein was also lost (**Figure S1G**), without changes in P3H1 mRNA level (data not shown). The expected loss of P3H1 protein was seen in fibroblasts from a patient with a homozygous frameshift mutation in *LEPRE1*, c.232delC, which results in loss of P3H1 mRNA [6] (**Figure S1K**) and was accompanied by loss of the CRTAP protein (**Figure S1I**); no effects were noted on CRTAP mRNA level (data not shown). These findings indicate that CRTAP and P3H1 are each essential components of the prolyl 3-hydroxylation complex, and that they interact for mutual stabilization.

## Discussion

Our recent studies and those from other groups highlighted the important role that CRTAP plays in bone mass homeostasis and recessive forms of OI [1], [4], [6], [9]. Although the usual clinical features that bring individuals with OI to clinical attention are fractures or other evidence of bone fragility, OI has many features of a multi-systemic disorder, and there is widespread expression of the major genes involved that express the two chains of type I procollagen. While bone, cartilage, tendon, and skin are the classical tissues affected in connective tissue disease, other organs, including lung and kidney, have a significant stromal component containing collagen fibrils and hence may also be affected. To gain additional insight into the pathophysiology of OI, we exploited the mouse model of inactivation of *Crtap*, a component of the prolyl 3-hydroxylation system important for type I procollagen processing. We found that CRTAP is produced in many extra-skeletal tissues that do not express very high levels of type I collagen, such as the lung and kidney. Most of these tissues analyzed from the *Crtap-/-* mice had mild alterations and an increase in cell proliferation, even with virtually complete lack of 3-hydroxylation of Pro 986 in chains of type I and II collagens, as previously described. The prolyl residue at the same position of the triple helical domain in the proα2(I) chain of type V collagen (encoded by *COL5A2*, a member of the same evolutionary clade as the type I and type II collagen genes) was similarly not hydroxylated. These data demonstrate both that CRTAP functions in tissues other than the skeleton and targets additional substrates that contribute to the ECM organization.

Type V collagen is a heterotrimeric, minor component of all type I collagen fibrils that contains several 3-hydroproline residues and helps regulate fibrillogenesis and fibril diameter of type I collagen [19], [20]. It is possible that the lack of 3-hydroxylation of this proline in type V collagen could contribute to the increased diameter of type I collagen fibrils observed in *Crtap-/-* mice [1]. Moreover, type V collagen is required to initiate collagen fibril assembly [21], and alteration of type V collagen could help mediate the striking decrease in osteoid volume in *Crtap-/-* mice [1]. Finally, mutations in the *COL5A1* and *COL5A2* genes that encode the chains of type V collagen cause Ehlers-Danlos syndrome types I and II [22], [23], which are characterized, among other defects, by skin laxity, a feature of the *Crtap-/-* mice. Skin laxity has also been observed in some patients with autosomal dominant OI forms resulting from type I procollagen gene mutations, and is a common finding in several connective tissue disorders [24]. Thus, the overlapping OI/Ehlers-Danlos features observed in *Crtap-/-* mice and in some patients with recessive forms of OI could be explained by the extended substrate for CRTAP and effects on prolyl 3-hydroxylation in collagens in addition to types I and II. Because prolyl 3-hydroxylation of chains of type IV collagen was normal, it is likely to be a substrate for a different hydroxylase (at least three are known in both mice and humans) and does not appear to depend on the presence of CRTAP. The morphological changes in the kidney therefore may instead be caused by abnormal collagen I and/or V expression or modification, although this remains to be proven. Additional functions of CRTAP cannot be excluded, unrelated to prolyl 3-hydroxylation, which may contribute to the tissue defects described here.

Our results have important clinical implications for patients with recessive OI caused by mutations in *CRTAP* or *LEPRE1*. The absence of both encoded proteins in patient fibroblasts when either the *CRTAP* or *LEPRE1* gene has a null mutation, most likely explains the overlapping skeletal phenotype seen in individuals with recessive OI that results from mutations in these genes [1], [4], [5], [6], [7], [8], [9]. It remains to be seen if these patients also have overlapping non-skeletal phenotypes, as it is unknown if CRTAP and P3H1 have functions that are dependent on one another in other tissues such as the lung and kidney. Reciprocal CRTAP/P3H1 co-stabilization has now been described by others and shown to be independent of CYPB expression [10], [25].

The phenotypes described in the *Crtap-/-* mouse lung, kidney, and skin may present as subclinical phenotypes in the human patients or, given the marked severity of the bony phenotype in most, may not have been studied. Regardless, our findings emphasize the importance of thorough monitoring of OI patients for non-skeletal consequences of their connective tissue disease. For instance, renal abnormalities are reported in OI patients that may not be detected without monitoring. In one series, 17 out of 47 individuals with OI had persistent hypercalciuria, correlating with severity of the OI, with one patient having isolated microscopic hematuria [26]; in a separate study 4 out of 58 OI children were found with nephrolithiasis [27]. It is likely that the large majority of these OI cases are due to mutations in the type I collagen genes, but it is unclear if the OI caused by *CRTAP*, *LEPRE1*, or *PPIB* mutations may also have renal abnormalities. Furthermore, it must be acknowledged that the hypercalciuria may be secondary to abnormal bone mineral metabolism, although our results suggest that a primary kidney defect should also be considered. Regardless, because individuals with OI are often supplemented with vitamin D and calcium, which are known to increase calcium flux in the kidney, it is especially important for the treating physician to be attentive to any renal complications. Glomerulopathy has been observed in another mouse model of osteogenesis imperfecta, the homotrimeric α1(I) collagen *oim/oim* mouse [28], suggesting that distinct molecular abnormalities in OI can result in a similar skeletal and extraskeletal phenotype. The subtle nature of this abnormality is confirmed by the absence of proteinuria by dipstick analysis in both our *Crtap-/-* mice (data not shown) and in the OI patients with hypercalciuria [24], as well as the normal levels of serum and urinary calcium, phosphorous, and magnesium previously reported in the *Crtap-/-* mice [1].

The most common causes of death in individuals with severe OI are respiratory problems, including pneumonia [13]. After exclusion of type II perinatal lethal OI, a pathological evaluation of the cause of death in 82% of the more severe OI type III and 39% of milder OI type I and OI type IV was considered respiratory [13]. Increasing scoliosis correlates with increasing restrictive lung disease [29]. However, the authors of that study note that “the presence of more severe restrictive lung disease with relatively smaller curve magnitudes in the population with OI indicates the possibility of intrinsic pulmonary abnormality” [29]. In addition, there are isolated reports of abnormal collagen in the lungs of individuals with severe OI [30], [31], [32]. Therefore, these data combined with our results suggest that a primary lung defect in individuals with OI may be a consequence of abnormal collagen synthesis, in addition to secondary consequences of skeletal abnormalities.

As with other connective tissue diseases, skin laxity and skin fragility have been observed in some individuals with OI, and it has long been known that cultured dermal fibroblasts from individuals with OI make abnormal proteins [33], [34]. Even if there is no obvious clinical skin abnormality, there can be alterations of the mechanical properties of the skin in OI, similar to that seen in the *Crtap-/-* mice [35]. Furthermore, recently described mice that are null for the *Ppib* gene, which encodes CYPB, also demonstrate skin laxity as well as weakness, suggesting that loss of components of the prolyl 3-hydroxylation complex may lead to common skin findings [36]. In addition, sections of the dermis of mice that are heterozygous for a null allele of *Col1a1* appear strikingly similar to the *Crtap-/-* skin sections [37]. These findings suggest that abnormal skin, albeit subclinical in most cases, may be a part of the phenotype of both classical OI and OI caused by mutations in *CRTAP* or *LEPRE1*.

In summary, absence of CRTAP in the mouse results in the pathophysiology of multiple organ systems, and CRTAP is required at the molecular level for proper prolyl 3-hydroxyation of several types of fibrillar collagen and for the expression of the P3H1 protein in humans.

## Materials and Methods

### Ethics Statement

All research involving animals was conducted according to relevant national and international guidelines. Trained veterinarians supervised animal care according to standard conditions of the Baylor College of Medicine (BCM) Center of Comparative Medicine (CCM). The BCM animal program is fully accredited by the Association for Assessment and Accreditation of Laboratory Animal Care International and is operated in compliance with the Guide for the Care and Use of Laboratory Animals. CCM also operates in coordination with the Institutional Animal Care and Use Committee whose membership and procedures comply with Public Health Service policy.

### Animal Tissue Collection and Processing

*Crtap-/-* mice and wildtype littermates were sacrificed, and lungs, kidneys, testes, and skin from the upper back were dissected, fixed, paraffin embedded, and sectioned according to standard methods as previously described [1]. Specifically, the skin was placed on Whatman filter paper immediately after dissection in order to maintain tissue integrity, and it was sectioned exactly perpendicular to the plane of embedding. The lungs of each mouse were inflated to a constant pressure of 25 cm with formalin fixative and then sutured closed at the trachea. The kidneys were cut longitudinally in order to ensure proper fixation. The *Crtap-/-* mouse colony was maintained in a mixed 129Sv/ev-C57Black/6J genetic background and housed in the Baylor College of Medicine Animal Vivarium.

### Histological Staining, Tissue Immunofluorescence, BrdU, and Apoptosis Assays

Standard protocols were followed for the following stains: Hematoxylin and Eosin, Toluidine Blue, and Picrosirius Red. Immunofluorescence on mouse tissues was done as previously described [38]. A rabbit polyclonal antibody raised against CRTAP protein was used. *Crtap-/-* tissues were used as a control for background and specificity of staining. Briefly, the paraffin sections were xylene treated, rehydrated, and heated for 20 minutes in a steamer for antigen retrieval. Subsequently they were incubated in blocking solution (3% normal Donkey serum, 0.1% BSA, 0.1% Triton X-100 in PBS), 1∶100 dilution of CRTAP antisera, 1∶600 donkey ant-rabbit secondary antibody conjugated to Alexa flour 594 (Invitrogen), and finally mounted with Prolong Gold anti-fade reagent with DAPI (Invitrogen). Cell proliferation status was assessed by BrdU incorporation using a Zymed BrdU labeling reagent kit and following the manufacturer recommendations. At 10 days post-natal growth, mice were injected with 10 mL/g of concentrated BrdU reagent two hours before tissue collection, and imaged using an anti-BrdU antibody conjugated to Alexa flour 488 (Invitrogen). Apoptotic cells were labeled with green fluorescent signal using the ApopTag Plus In Situ Apoptosis Flourescein Detection kit (Millipore). At the end of each described procedure, images were captured using a Zeiss Axioplan 2 microscope.

### Lung Morphometry

The mean linear intercept (MLI) method was used to obtain quantitative analysis of the distal airway space enlargement, as previously described [39]. At least 10 histological fields per mouse were captured at 20X magnification from all lobes of both lungs. The ImageJ software grid analysis plug-in (rsb.info.nih.gov/ij) was used to overlay a grid consisting of 10 horizontal lines and 14 vertical lines onto each 521 micrometer X 697 micrometer image. Intersections of alveolar walls with each grid line were manually counted, and the MLI was calculated as the total length of lines analyzed divided by the total number of intercepts counted.

### Electron Microscopy

Freshly dissected tissues were fixed in 1.5% glutaraldehyde/1.5% paraformaldehyde with 0.05% tannic acid in 0.1 M Cacodylate buffer, pH 7.4 for 60 minutes on ice, rinsed in 0.1 M cacocylate overnight, then postfixed for 60 minutes in cacodylate buffered 1% OsO4, rinsed, then dehydrated in a graded ethanol series from 30–100%. The samples were washed in propylene oxide and embedded in Spurrs epoxy. Ultrathin sections were stained in Uranyl Acetate followed by Reynolds lead citrate and examined using a FEI Tecnai G2 TEM.

### Skin Tension Test

Samples were prepared from the dorsal skin of adult (9 month-old) wildtype and *Crtap-/-* male mice (n = 3). The skin was harvested and cut into 1 cm wide by 4 cm long pieces. The long axis of the sample coincided with the superior–inferior direction of the mouse. The samples were clamped between two aluminum plates at the superior and inferior ends of the sample. Tension tests were performed on a MTS (Eden Prairie, MN) servohydraulic materials test machine. The samples were preloaded to 0.2 Newtons and then loaded to failure in tension at a constant rate of 10 mm/min. Peak load and stiffness were recorded using TestWorks 4 software (Eden Prairie, MN).

### Preparation of collagens

Types I and V collagens were prepared from WT and *Crtap-/-* bone. Bone was defatted at 4°C in 0.5 M EDTA, 0.05 M Tris-HCl, pH 7.5. Type IV collagen was prepared from WT and *Crtap-/-* kidney. The kidney was equilibrated in saline containing protease inhibitors. Tissues were finely minced and collagens solubilized by pepsin (1∶20, w/w, pepsin/dry tissue) in 3% acetic acid for 24 h at 4°C. Bone collagens I and V were serially precipitated at 0.7 and 1.8 M NaCl respectively. Kidney collagens were serially precipitated at 0.7 and 1.8 M NaCl and type IV collagen was reprecipitated from the 1.8 M fraction at 2.5 M NaCl after dissolving in 1 M NaCl, 0.05 M Tris-HCl, pH 7.4. For SDS-PAGE, the method of Laemmli was used with 6% gels for pepsinized collagen.

### Mass spectrometry

Collagen α-chains were cut from SDS-PAGE gels and subjected to in-gel trypsin digestion. Electrospray MS was performed on the tryptic peptides using an LCQ Deca XP ion-trap mass spectrometer equipped with in-line liquid chromatography (LC) (ThermoFinnigan) using a C8 capillary column (300 µm×150 mm; Grace Vydac 208 MS5.315) eluted at 4.5 µl min. Sequest search software (ThermoFinnigan) was used for peptide identification using the NCBI protein database.

### Human Cell Culture and Immunofluorescence

Primary human fibroblasts were cultured in DMEM with 4 mM L-glutamine and 4500 mg/L glucose (HyClone) plus 10% FBS, 100 units/mL penicillin, and 100 micrograms/mL streptomycin. The cells were split into glass LAB-TEK 4-well chamber slides (Nunc), and 24 hours later were fixed with 4% paraformaldehyde, treated with 0.1% Triton X-100, blocked in 10% donkey serum and 1% BSA, and then sequentially incubated with 1∶250 dilution of CRTAP antisera or P3H1 mouse MaxPab polyclonal antibody (Abnova), 1∶500 donkey anti-rabbit secondary antibody or donkey anti-mouse secondary antibody conjugated to Alexa Flour 594 (Invitrogen), and mounted with Prolong Gold anti-fade reagent with DAPI (Invitrogen). The cells were also co-stained with Phalloidin conjugated to Alexa Flour 488 (Invitrogen) in order to visualize the actin cytoskeleton and the overall cell outline.

### Statistical Analysis

Student's t-test was used to identify statistically significant differences, with p<0.05 considered significant. All bar graphs display the mean, plus or minus the standard error of the mean.

## Supporting Information

Figure S1 Immunofluorescence of CRTAP and P3H1 protein in control primary human fibroblasts and in patients with recessive osteogenesis imperfecta due to mutations in *CRTAP* or *LEPRE1* (which codes for P3H1). In the control fibroblasts, the CRTAP (A) and P3H1 (C) staining patterns are each consistent with ER localization. In *CRTAP* mutant cells, there is loss of staining of both CRTAP (E) and P3H1 (G). In *LEPRE1* mutant cells, there is also loss of staining of both CRTAP (I) and P3H1 (K). Panels (B), (D), (F), (H), (J), and (L) are merges of CRTAP or P3H1 staining with green fluorescent labeled phalloidin and DAPI to show cellular morphology. All images are at 20X magnification.
(3.05 MB TIF)
