# Supplementary figures and images for: Generalized Connective Tissue Disease in Crtap-/- Mouse
Source: PLoS One. 2010 May 11;5(5):e10560. doi: 10.1371/journal.pone.0010560 (PMC2868021; doi:10.1371/journal.pone.0010560)

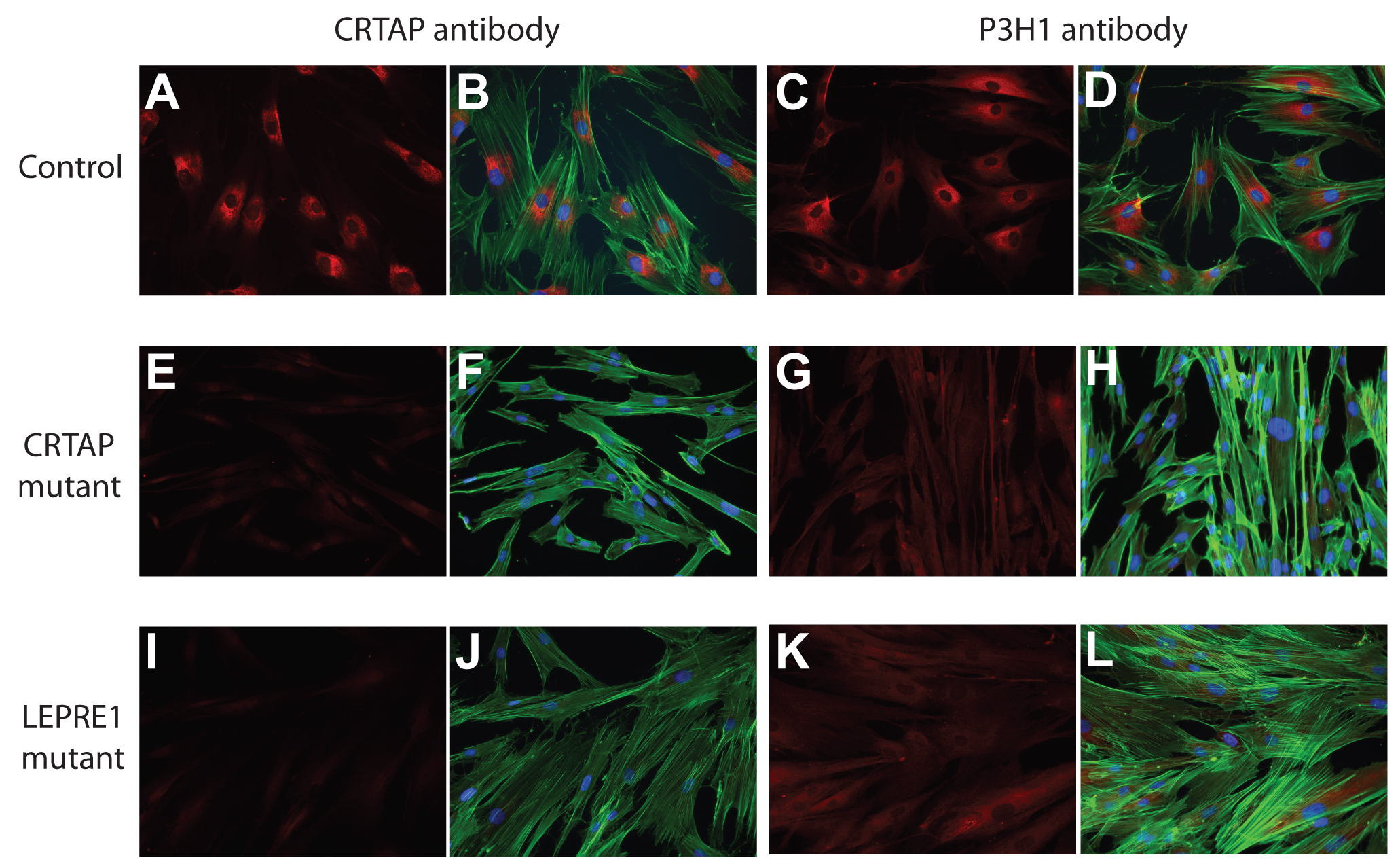

Supplement: Figure S1 — Immunofluorescence of CRTAP and P3H1 protein in control primary human fibroblasts and in patients with recessive osteogenesis imperfecta due to mutations in CRTAP or LEPRE1 (which codes for P3H1). In the control fibroblasts, the CRTAP (A) and P3H1 (C) staining patterns are each consistent with ER localization. In CRTAP mutant cells, there is loss of staining of both CRTAP (E) and P3H1 (G). In LEPRE1 mutant cells, there is also loss of staining of both CRTAP (I) and P3H1 (K). Panels (B), (D), (F), (H), (J), and (L) are merges of CRTAP or P3H1 staining with green fluorescent labeled phalloidin and DAPI to show cellular morphology. All images are at 20X magnification. (3.05 MB TIF) [file pone.0010560.s001.tif]
